# Risks of Type 2 diabetes among older people living with HIV: A scoping review

**DOI:** 10.4102/safp.v65i1.5623

**Published:** 2023-05-23

**Authors:** Nongiwe L. Mhlanga, Thinavhuyo R. Netangaheni

**Affiliations:** 1 Department of Health Studies, College of Human Sciences, University of South Africa, Pretoria, South Africa

**Keywords:** people living with HIV, Type 2 diabetes, risks, HIV, older

## Abstract

**Background:**

The effectiveness of antiretroviral therapy has enabled people living with human immunodeficiency virus (HIV) to live longer. With this longevity, there is the risk of developing age-related chronic conditions like Type 2 diabetes. Older people living with HIV have an increased risk of Type 2 diabetes mellitus (Type 2 DM) because of the natural physiological processes of ageing and risks posed by HIV infection and antiretroviral therapy use. The purpose of this scoping review is to describe risk factors associated with the development of Type 2 DM among older people living with HIV.

**Methods:**

The study adopted a framework for scoping reviews. The first step identified the research question, followed by identifying studies from three databases: PubMed, Mendeley and Cochrane Library. A total of 618 nonduplicate studies were screened, with a final selection of 15 full-text studies from 2012 to 2022. Data were extracted using the Souza (2010) data extraction tool and analysed numerically and with thematic content analysis.

**Results:**

Most studies were from Italy, with 60% being cross-sectional studies. On thematic analysis, the risks associated with Type 2 DM among older people living with HIV were long duration of HIV infection; the use of older-generation antiretroviral therapy; a high body mass index; the presence of hypertension and a lack of knowledge on modifiable risk factors for Type 2 DM.

**Conclusion:**

The identification of the risks that increase the likelihood of Type 2 DM among older people living with HIV facilitates effective screening and focused health education for older people living with HIV to reduce the development of Type 2 DM.

**Contribution:**

The findings of this study of excess risks of Type 2 DM specific to older people living with HIV complement risk factors of Type 2 DM in the general population. These excess risks facilitate case finding of older people living with HIV at risk of Type 2 DM especially in primary healthcare settings.

## Background

Human immunodeficiency virus (HIV) infection is a chronic condition affecting the body’s immune system (primarily cluster of differentiation 4 [CD4] cells) and was first described in the United States (US) in the early eighties.^1^ By the end of 2021, at least 38.4 million people had been living with HIV,^2^ with at least 20.6m people living with HIV in Eastern and Southern Africa.^3^ Treatment of HIV entails the use of antiretroviral therapy (ART), introduced in the mid-nineties. Four decades after this description of HIV, at least 28.7 m of all people living with HIV are accessing ART^2^; people living with HIV are living longer lives as a result of the effectiveness of ART.^1^ The Association of Nurses in AIDS (acquired immunodeficiency syndrome) Care (ANAC)^4^ estimates that by 2030, an estimated 70% of all people living with HIV globally will have reached the age of 50 years. With this longevity, there is the risk of acquiring noncommunicable chronic conditions associated with ageing like Type 2 diabetes mellitus (Type 2 DM).^4^ Type 2 DM is an age-related condition characterised by high blood glucose because of insulin resistance or the body’s failure to produce enough insulin.^5^ Type 2 DM accounts for 90% of all cases of diabetes.^5^ The World Health Organization (WHO)^5^ notes that by 2019, diabetes was the ninth leading cause of death globally, with hypertension being the lead cause.^6^ The WHO^5,6^ also notes that most people affected by Type 2 DM and hypertension reside in developing countries.

Among people living with HIV, estimates of prevalence of Type 2 DM vary across regions. Chencinar and Barber^7^ note that Type 2 DM among people living with HIV may be up to four times higher than in the general population in some regions. In a study conducted in the US, Type 2 DM among people living with HIV was estimated at 15%.^8^ Closer to this, in Saudi Arabia, Type 2 DM was the most common noncommunicable disease affecting people living with HIV, with at least 15.4% of all people living with HIV having Type 2 DM.^9^ In sub-Saharan Africa, prevalence of hyperglycaemia among people living with HIV ranges from 1% to 26%.^7^ Husain et al.^10^ concur with these results and reveal that Type 2 DM among people living with HIV in Africa is estimated between 2.1% and 25.5%. In Southeast Asia, one study found that the incidence of Type 2 DM among people living with HIV was 11.0 per 1000 person-years of follow-up.^11^ A closer incidence rate to Southeast Asia was found in a European study by Galaviz et al.,^12^ in a study in Finland of a higher incidence of 9.5 per 1000 person-years among men living with HIV compared with 7.0 per 1000 person-years of follow-up in men in the general population.

Several risks have been identified that increase likelihood of developing Type DM among people living with HIV. Firstly, people living with HIV face the same risks as the general population who develop Type 2 DM.^13^ These risks include a lack of physical activity, smoking, older age, urban dwelling, family history of diabetes, obesity and unhealthy dietary habits.^12^ Secondly, in addition to these risks, HIV infection and the use of ART also increase likelihood of Type 2 DM.^12^ Husain et al.^10^ explain the risks in people living with HIV by noting that Type 2 DM in people living with HIV is associated with lipodystrophy and chronic inflammation.

Unique to older people living with HIV, Leroith et al.^14^ note that there is an acceleration in the development of Type 2 DM because of common pathways associated with ageing and those associated with HIV infection. The authors^14^ reveal that the pathophysiology of Type 2 DM is worsened by metabolic changes that occur because of ageing. These mechanisms that increase the likelihood of Type 2 DM with ageing are an increase in body fat composition, changes in body fat distribution and a decline in lean mass.^15^ On the other hand, HIV infection and the use of ART heighten the risk of Type 2 DM through HIV-related chronic inflammation and lipodystrophy.^13^

Despite these excess risks of developing Type 2 DM in older people living with HIV, a research gap in the population exists where older people living with HIV are under-investigated for risks of developing Type 2 DM. Miles^16^ defines such a research gap in the population as ‘research undertaken when a population is inadequately presented in previous research’. This scoping review is important because the identification of risks for the development of Type 2 DM among older people living with HIV enables the implementation of interventions to reduce the incidence of Type 2 DM among older people living with HIV. Therefore, the rationale of this scoping review of literature is to describe risk factors associated with the development of Type 2 DM among older people living with HIV.

Before conducting the scoping review, the objectives, inclusion criteria and methods of analysis for this review were specified in advance and documented in a protocol. A preliminary search of Cochrane Library, PubMed and Mendeley databases was conducted for a scoping review with a topic similar to this study, and no matching results were found.

## The objective of the study

The objective of the scoping review is to describe the risks associated with the development of Type 2 DM among older people living with HIV.

### Research questions

- What research approaches and designs have been used to identify Type 2 DM risks among older people living with HIV?
  Rationale: No scoping review study design was found on three databases (Cochrane Library, PubMed and Mendeley) on risks associated with the development of Type 2 DM among older people living with HIV.
- In which settings (socio-economic, geographic and healthcare) have research on risks associated with the development of Type 2 DM among older people living with HIV been conducted?
  Rationale: Study findings are reported in the context of geographical, socio-economic and healthcare settings, as outcomes vary according to the different contexts.
- What are the population age characteristics of studies conducted on risks of developing Type 2 DM among people living with HIV?
  Rationale: Distinguishing older and younger people enables the identification of risks specific to older people living with HIV.
- What are the risks associated with the development of Type 2 DM among older people living with HIV?
  Rationale: Identifying risks associated with Type 2 DM enables the implementation of interventions to reduce the occurrence of Type 2 DM among older people living with HIV.

## Methods

This study utilised a scoping review methodology defined by Levac et al.^17^ as a qualitative method of knowledge synthesis that addresses an exploratory research question with the aim of mapping key concepts, types of evidence and gaps in research related to a particular area of research by methodically searching, selecting and synthesising existing knowledge. Three issues are critical in conducting scoping reviews, namely the population, the context and the concept.^18^ In this study, a scoping review was performed to provide a ‘concept map’ that will answer the research objective.

The study adopted the framework for conducting scoping reviews from Arksey and O’Malley, which was updated by Peters et al.^19^ The framework uses six steps, which include: (1) identifying the research question; (2) identifying the studies; (3) selecting the studies; (4) charting the data; (5) collating, summarising and reporting findings; and a sixth optional step of consultation, which was not included in this study.^19^

### Step 1: Identifying the research question

To identify the research question a preliminary literature review was done of risks of Type 2 DM among older people living with HIV. This review provided useful insight into some factors that predispose a person to Type 2 DM, such as genetics, which are not age dependent. This insight on factors associated with Type 2 DM provided clarity on risks affecting older people living with HIV aiding in refining the research question to ‘what are the risk factors associated with the development of Type 2 DM among older people living with HIV?’ This refinement aided in the selection of studies.

### Step 2: Identifying relevant studies

The second step after the identification of the relevant research question is the selection of relevant studies to include in the search.^19^ Published literature was drawn from three databases, PubMed, Mendeley and the Cochrane Library. These databases were selected as they provided literature from different health disciplines. The selection of studies considered the context, the concepts and the population. Keywords for the selection of studies were ‘diabetes’, ‘risks’, ‘people living with HIV’. The literature reviewed was from 2012 to 2022 and was published in English. Studies that were not in English were excluded because of the time and cost involved in the translation and the risk of distortion in translation. The studies identified from the three databases were 996, and duplications were removed using the Mendeley Reference Manager. The literature search was conducted from 15 April 2022 to 13 July 2022, with follow-up performed until the 31 August 2022. Grey literature was searched through targeted websites such as the WHO and the International Diabetes Federation, and no results were found.

### Step 3: Selecting the studies

The inclusion and exclusion criteria detailed the studies to be considered, and these studies included the key words of ‘people living with HIV’, ‘risks’ and ‘Type 2 DM’. To select these studies, articles were selected based on the following inclusion criteria:

- Studies had to include risk factors associated with Type 2 DM in people living with HIV.
- ‘Ageing’, ‘older people’ or ‘persons more than 50 years’ had to be included as a variable in determining these risks associated with Type 2 DM.
- Studies had to be original research.

Literature was excluded based on the following criterion:

- studies that did not include ‘ageing’ as a variable in determining risks associated with Type 2 DM
- studies which were not original research.

A team of reviewers reviewed selected sources to ensure the selection was comprehensive.^18^ Three steps were involved in selecting the studies: the first was a review of all titles and abstracts identified in the search by the first author; the second author then reviewed a minimum of 10% of the articles selected by the first author until an agreement was reached on the number of selected articles, based on the inclusion and exclusion criteria. The second step involved the review of all articles selected by both authors and the application of the inclusion and exclusion criteria by the first author, which were all articles that included ‘ageing’ as a variable. The second author then reviewed at least 10% of the selected articles after review by the first author. The third step was the first author referring all the articles they were unsure about including to the second author for review and a final check.

### Step 4: Charting the data

The fourth step in conducting a scoping review of literature is charting the data.^18^ Peters et al.^19^ note that the researcher will go to each paper and extract information that is relevant to the aim of the study. The Souza data extraction tool was used to extract data, and this included the authors, title, year of publication, country of origin, purpose, research objectives, research designs, sampling and population, key concepts and results that relate to the study objective.^20^ Data were charted using Microsoft Excel (Microsoft Corporation, Redmond, Washington, United States).

### Step 5: Collating, summarising and reporting findings

The fifth step was collating study findings and discussing the implications for further research.^18^ This step involved three main steps: the first step was a basic numeric analysis which highlighted the years of study, the origins, the study designs and the context in which the studies took place. The second step was thematic content analysis. The themes extracted pertained to the risk factors for the development of Type 2 DM among older people living with HIV. The third and last step was describing the implications of the findings for practice, future research, education and policy.^19^

### Ethical considerations

Ethical approval to conduct the study was obtained form the Research Ethics Committee of the University of South Africa (ref. no. 14056739_CREC_CHS_2022).

## Results

**FIGURE 1:**
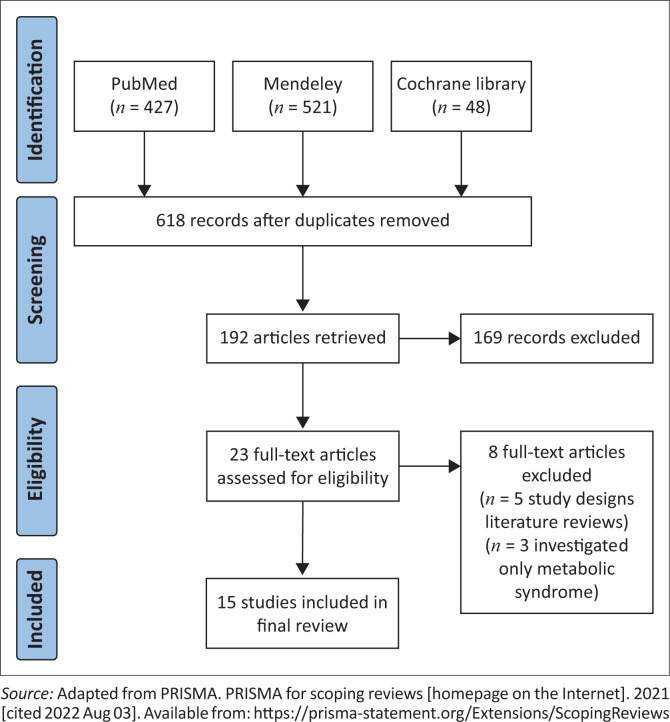
Preferred Reporting Items for Systematic Reviews and Meta-Analysis flow chart of the flow of studies at each phase of the scoping review process.

The three database searches yielded a total of 996 studies; duplicates were then removed using the Mendeley Reference Manager to yield 618 records. From these, the inclusion and exclusion criteria were applied to yield 192 full-text articles. From the 192 articles, the inclusion and exclusion criteria were reapplied, which excluded articles that did not include ‘ageing’ of people living with HIV. From these, 23 articles remained and eight were then excluded, because five were literature reviews that did not define the population which was of key interest, and three focused on metabolic syndrome only and did not include Type 2 DM. The final screening for eligibility yielded 15 articles. The Preferred Reporting Items for Systematic Reviews and Meta-Analysis (PRISMA) flow chart in Figure 1 shows the decision process.

### Characteristics of the included studies

Numerical data for the findings were analysed using Microsoft Excel, which highlighted the years of the studies, the types of studies and the geographical coverage of the studies. A total of 15 studies were selected from the literature. The articles selected were from the years 2012 to 2022. Table 1 shows the years in which the articles retrieved were published.

**TABLE 1:** Years of studies.

Year	Number of studies (*n* = 15)	Percentage
2012	1	6.67
2016	1	6.67
2017	3	26.67
2018	3	20.00
2019	3	20.00
2021	3	20.00
2022	1	6.67

Three (20%) of the studies reviewed were from 2017.^22,23,24^ Another three studies^25,26,27^ (20%) were from 2018, and another three^28,29,30^ (20%) were from 2019. An additional three (20.00%) studies^31,32,33^ were from 2021. One study^34^ (6.67%) reviewed was from 2012, another (6.67%) study^35^ was from 2016 and one (6.67%) study^36^ was from 2022. No studies were retrieved from 2013, 2014, 2015 and 2020.

All studies reviewed were quantitative studies and used different study designs. Table 2 shows the study designs.

**TABLE 2:** Study design.

Study design	Number of studies (*n* = 15)	Percentage
Cross-sectional study	9	60.00
Secondary data analysis	2	13.33
Nonintervention cohort study	1	6.67
Regional observational cohort design	1	6.67
Cohort study	1	6.67
Match control study	1	6.67

The majority of the studies (60.0%) were cross-sectional studies,^23,25,26,27,28,31,32,34,35^ while 13.33% of the studies^24,33^ were secondary data analysis. Nonintervention cohort studies,^36^ regional observational studies,^30^ cohort studies^22^ and match control studies^29^ consisted of 6.67% of each of the total studies reviewed.

With regards to the place of origin of the studies, Table 3 outlines where the studies were conducted.

**TABLE 3:** Place of origin of studies.

Place of origin	Number of studies (*n* = 15)	Percentage (%)
Italy	3	20.00
Zimbabwe	2	13.30
Italy and Romania	1	6.67
Asia and Pacific	1	6.67
United Kingdom	1	6.67
Denmark	1	6.67
Tanzania	1	6.67
Rwanda	1	6.67
South Africa	1	6.67
Canada	1	6.67
United States	1	6.67
Brazil	1	6.67

The majority of the studies (20%) were conducted in Italy,^22,26,34^ while 13.3% were conducted in Zimbabwe.^23,32^ One study (6.67%) drew participants from Italy and Romania.^29^ Another study^30^ (6.67%) was a multicountry study from the Asia and Pacific region where data were taken from China, Cambodia, Hong Kong Special Administrative Region, Indonesia, India, Malaysia, Japan, the Philippines, Singapore, South Korea, Taiwan, Vietnam and Thailand. The remaining studies reviewed were from the United Kingdom (UK)^25^ (6.67%), Denmark^36^ (6.67%), Tanzania^27^ (6.67%), Rwanda^28^ (6.67%), South Africa^33^ (6.67%), Canada^24^ (6.67%), the US^32^ (6.67%) and Brazil^31^ (6.67%).

The population of interest in the study was older people living with HIV. From the 15 studies selected, the sample sizes ranged from 82 to 13 397. All studies included people living with HIV, and one match-control study from Italy^29^ included children who had acquired HIV prenatally; this study also included older people and geriatric patients who were living with HIV aged more than 50 years and were assessed between the ages of 75 and 80 years. Two studies^26,36^ used only respondents aged more than 65 years and more than 50 years, respectively.

In terms of the study participants’ dwellings, two studies^27,28^ from Africa reported collecting data from rural locations. One study^32^ noted that the study respondents were from low socio-economic status. One study^25^ in the UK also noted the diversity of the sample by revealing that respondents were residents of London but were composed of persons from 61 different countries, which represents the diversity of the population in the area. Table 4 summarises the study populations of the selected studies.

**TABLE 4:** Study population.

Authors	Sample size	Population characteristics
Duncan et al.,^25^ UK	First cohort (2005) 337, second cohort (2015) 338	The older second cohort in 2015 had a median age of 49 years, the 2005 cohort a median age of 41 years. people living with HIV recruited from three London clinics.
Cassenote et al.,^31^ Brazil	6727	The adult population older than 18 years followed from 2003 to 2016, taken from the Brazilian HIV and AIDS cohort study from four of five administrative regions of the country.
Da Luca et al.,^22^ Italy	6505	Patients enrolled in ICONA prospective study, followed until September 2014.
Chimbetete et al.,^23^ Zimbabwe	4110	People living with HIV followed from 01 March 2004 to 29 April 2015 aged more than 16 years. Recruited from Newlands Clinic Harare.
Høgh et al.,^36^ Denmark	643	People living with HIV aged 18 years from the Greater Copenhagen area. Baseline data were collected from March 2015 to April 2017. Follow-up was carried out from April 2017 to April 2019.
Tiozzo et al.,^32^ US	82	People living with HIV 18 years and above, mean age 48 years, from the Adult HIV Clinic at the University of Miami (UM) Miller School of Medicine–Jackson Health Care System and other Miami–Dade County clinics.
Magodoro et al.,^35^ Zimbabwe	1033	People living with HIV aged 18 and above, median age 42 years, from Mpilo HIV Clinic.
Guaraldi et al.,^26^ Italy	1573	1258 people living with HIV, 315 HIV-negative participants who were followed from June 2015 to May 2016 from 10 clinics in Northern, Central and Southern Italy.
Kagaruki et al.,^27^ Tanzania	671	People living with HIV aged 18 years and above from Mbeya and Dar es Salaam rural and urban settings, respectively.
Biraguma et al.,^28^ Rwanda	794	People living with HIV aged 18 years and above, mean age 37.5 years, from Kigali city, northern and southern provinces.
Chiwandire et al.,^33^ South Africa	2005: 978; 2008: 1023; 2017: 2483	People living with HIV aged 25 years and above were selected from the Southern African National HIV Prevalence Incidence Behaviour Communication Surveys in 2005, 2008 and 2017.
Samad et al.,^24^ Canada	1065	Data from St Paul’s Hospital, Vancouver, British Columbia, older than 50 years, followed for a median age of 13 years.
Guaraldi et al.,^29^ Italy	131 young; 32 old; 77 geriatric	People living with HIV taken from Modena HIV Metabolic Clinic and the Romanian HIV Clinic. All patients had been living with HIV for more than 25 years.
Han et al.,^30^ Asia and Pacific Region	1927	Used the TREAT Asia HIV Observational Database, recruited from 2003 to 2017 from 20 sites. Median age 35 years.
Galli et al.,^34^ Italy	4249 people living with HIV and 9148 HIV-negative control group	People living with HIV recruited from San Raffaele Infectious Diseases Department, the control group was recruited from 15 regions in Italy.

HIV, human immunodeficiency syndrome; AIDS, acquired immunodeficiency syndrome; ICONA, Italian Cohort Naïve Antiretrovirals; TREAT, Therapeutics Research, Education and AIDS Training in Asia; US, United States; UK, United Kingdom.

## Emerging themes: Risk factors associated with the development of Type 2 DM among older people living with HIV

The 15 articles reviewed highlighted the risk factors associated with the development of Type 2 DM among older people living with HIV. The main themes and subthemes that emerged from the scoping review were (1) duration of HIV infection, (1.1) duration of HIV infection and a low CD4 nadir, (2) use of older-generation ART, (2.1) adverse effects of ART in older people living with HIV, (3) presence of hypertension, (4) a high body mass index (BMI) among older people living with HIV and (5) a lack of knowledge on modifiable risks of Type 2 DM among older people living with HIV.

### Duration of HIV infection in older people living with HIV is associated with Type 2 DM

The first theme identifying the risk of Type 2 DM among older people living with HIV was the duration of HIV infection. The issue of long duration of HIV infection was noted in the findings of seven studies. Duncan, Goff and Peters,^25^ in their study findings from a cross-sectional study in the UK, used a 2005 cohort and a 2015 cohort which was much older, with a mean age of 49 years compared to 41 years in the 2005 cohort. In their findings, the authors^25^ reported a higher prevalence of Type 2 DM (32.3%) among the older 2015 cohort and attributed this to a ‘longer known duration of HIV infection’. The risk posed by a long duration of HIV infection and ageing is also described by a study conducted in Italy by Guaraldi et al.,^26^ who found a higher prevalence of Type 2 DM among people living with HIV longer than 20 years. Notably, the respondents in this study^26^ were more than 65 years of age (mean age 71 years among women and 72 years among men) and were compared to a cohort who were HIV-negative. In a later match-control study, Guaraldi et al.^29^ used a sample from Italy which was older and a sample of people living with HIV who acquired HIV prenatally from Romania and concluded that HIV infection modulates the age at which people living with HIV acquire comorbidities, inclusive of Type 2 DM. The study^29^ followed three control groups for 25 years: those who acquired HIV prenatally, those who acquired HIV after the age of 25 and those who acquired HIV at 50 years; the study concluded that a longer HIV duration in people who acquired HIV after the age of 50 years predisposes them to comorbidities such as Type 2 DM.^29^ Notably, the conclusion made by Guaraldi et al.^29^ that acquisition of HIV infection after the age of 50 increases the likelihood of Type 2 DM is in contrast to findings by Samad et al.,^24^ who found that there is no association between incident Type 2 DM and HIV acquisition before or after the age of 50 years. In Italy, Galli et al.^34^ in a cross-sectional study also concluded that Type 2 DM was associated with older age and longer duration of HIV infection. The risk posed by a long duration of HIV infection to the development of Type 2 DM is also highlighted by Høgh et al.,^36^ in a study in Denmark with respondents drawn from the Copenhagen area with a mean age of 52 years, noted that insulin resistance was associated with duration of HIV infection and increased with every 5 years. This issue of the long duration of HIV infection increasing the likelihood of Type 2 DM is further elaborated by Cassenote et al.,^31^ in a study in Brazil, which found that the mean time to developing Type 2 DM was 10.5 years with a median age of 11.8 years. Samad et al.^24^ in Canada also concluded that there was an association between the development of Type 2 DM and a long duration of HIV infection among older people living with HIV; the authors^24^ found that the mean time to development of Type 2 DM among older people living with HIV was 9 years (range 6.2–11.6 years). In their study,^24^ the authors used a sample of people living with HIV above the age of 50 years.

In the US, Tiozzo et al.,^32^ in an analytical cross-sectional survey, used a sample with a mean age of 48 years and also reported that a ‘longer duration of HIV; consequentially ageing’ is associated with risk of Type 2 DM. The study^32^ defined a longer duration of HIV infection as longer than 16 years and noted that the majority (59%) of their respondents, who were older than 50 years and had a longer duration of HIV infection, were most likely to develop Type 2 DM. In Africa, one study^35^ from Zimbabwe also noted that a long duration of HIV infection predisposes patients to Type 2 DM. In this cross-sectional study conducted by Magodoro et al.,^35^ a sample from Mpilo Central Hospital of 1225 people living with HIV concluded that the duration of HIV infection longer than 7 years was associated with common comorbidities such as Type 2 DM. The authors^35^ further note, like Guaraldi et al.,^29^ that age above 50 years and a long duration of HIV infection are associated with the likelihood of Type 2 DM.

### Lower CD4 cell nadir and long duration of HIV are associated with the development of Type 2 DM

A subtheme to emerge on the duration of HIV infection was that a lower nadir CD4 is associated with Type 2 DM.

The study by Høgh et al.^36^ also associated the risk of insulin resistance with a long duration of HIV and a lower CD4 cell nadir. The authors^36^ noted the risk of insulin resistance is increased among people living with HIV who had a lower CD4+ cell nadir of less than 200 cells/mm^3^. In Italy, Galli et al.,^34^ who used a sample of people living with HIV from the St Raffaele Infectious Diseases Department, also noted the high incidence of Type 2 DM among people living with HIV who had a lower nadir CD4 and a long duration of HIV infection. Samad et al.,^24^ concurring with these studies^34,36^ conducted from the St Raffaele Infectious Diseases Department and greater Copenhagen area, found that Type 2 DM was related to a history of a lower CD4 cell nadir of less than 80 cells/mm^3^, a viral load more than 500 copies/mm^3^ and a long duration of HIV infection.

### Use of older-generation antiretroviral therapy is associated with Type 2 DM among older people living with HIV

The second main theme emerging from the studies reviewed is the issue of adverse effects of ART on older people living with HIV predisposed to Type 2 DM. From this theme, subtheme of adverse effects of some ART increases likelihood of Type 2 DM.

The study conducted in Canada by Samad et al.,^24^ who used a secondary data analysis method from the outpatient HIV clinic at St Paul’s Hospital in Vancouver, reported that Type 2 DM was associated with ART initiation between 1997 and 2004. In their study, the authors^24^ compared this likelihood of developing Type 2 DM with people living with HIV who initiated ART between 2010 and 2015 who had a lesser incidence of Type 2 DM. The authors^24^ also noted that Type 2 DM was associated with a longer duration on ART as well as having commenced ART in an ‘earlier calendar year’. Similar to this study^24^ in Canada, Høgh et al.^36^ used a 2-year cross-sectional study design with respondents from March 2015 through December 2016 with follow-up from April 2017 to April 2019, and they found that previous use of older-generation ART was also associated with the development of Type 2 DM. The study^36^ defined older-generation ART as the thymidine analogues of stavudine, zidovudine and didanosine. Both studies by Samad et al.^24^ and Høgh et al.^36^ further elaborate that older-generation ART was associated with abdominal obesity, which is also a risk factor for developing Type 2 DM. Also concurring with Samad et al.^24^ and Høgh et al.,^36^ Guaraldi et al.^26^ in Italy in a study with respondents aged more than 65 years revealed an association between Type 2 DM and first-generation ART use. Guaraldi et al.^26^ also explained the cause of this risk of exposure to older-generation ART by revealing that older-generation ART caused lipodystrophy. Studies by Høgh et al.,^36^ Guaraldi et al.^26^ and Samad et al.,^24^ in addition to concluding that older-generation ART is associated with the development of Type 2 DM, also associate Type 2 DM with long duration of HIV infection.

### Adverse effects of ART among older people living with HIV are associated with Type 2 DM

The study also found that some classes of ART used in older people living with HIV were associated with the development of Type 2 DM. Cassenote et al.,^31^ in a study in Brazil with 6727 respondents drawn from four out of five regions of the country, found that exposure to some nucleoside reverse transcriptase inhibitors (NRTIs) in older people living with HIV is associated with the incidence of Type 2 DM. The authors^31^ highlighted the likelihood of Type 2 DM among people living with HIV between the ages of 40 and 50 years and those aged more than 50 years. The study^31^ also found that exposure to stavudine for longer than 2 years was associated with incident Type DM in older people living with HIV. Concurring with Cassenote et al.,^31^ De Luca et al.,^22^ in a multicentre study of 42 tertiary healthcare facilities in Italy conducted with an almost similar sample size of 6505, found that diabetes could be predicted among older people who were treated with regimens containing an NRTI (stavudine and lamivudine) and an ‘unboosted’ protease inhibitor. Samad et al.,^24^ albeit using a smaller sample size of 703, also noted that older people living with HIV had a higher incidence of Type 2 DM with the use of protease inhibitors in comparison to non-nucleoside reverse transcriptase inhibitors (NNRTIs). In their study, the authors^24^ also explained that exposure to stavudine, didanosine, zidovudine, nelfinavir, indinavir and lopinavir was associated with the development of Type 2 DM among people living with HIV older than 50 years.

The adverse effects of ART use in older people living with HIV were also reiterated by Han et al.,^30^ in a study in the Asia and Pacific region who, albeit using a sample with a mean age of 35, noted that ART initiation after the between ages 41 and 50 years and more than 50 years was associated with the development of Type 2 DM. Similarly, Duncan, Goff and Peters^25^ noted that weight gain after ART initiation in older people living with HIV was associated with Type 2 DM.

### High body mass index is associated with the development of Type 2 DM among older people living with HIV

From the studies reviewed, three studies found an association between a high BMI among older people living with HIV and the development of Type 2 DM.

In the UK, Duncan, Goff and Peters^25^ found that Type 2 DM prevalence was higher in their second older (mean age 49 years) cohort assessed in 2015 who had a higher BMI of 27.4 kg/m^2^. In their description of the 2015 cohort, Duncan, Goff and Peters^25^ revealed that the older cohort was heavier and had lower rates of lipodystrophy. Høgh et al.,^36^ in their study in Denmark, also found a higher incidence of insulin resistance among people living with HIV who had a BMI between 25 kg/m^2^ and 29 kg/m^2^ and who had central obesity. Guaraldi et al.^26^ also associated the likelihood of multimorbidity inclusive of Type 2 DM with a higher BMI and an age above 75 years. The authors^26^ described the risk posed by a high BMI as an independent predictor of Type 2 DM. Similarly, the study^23^ in Zimbabwe also discussed that a higher incidence of Type 2 DM in people living with HIV older than 50 years was associated with an increase in age and a higher BMI. On the contrary, Samad et al.^24^ concluded that the likelihood of Type 2 DM was also found among people living with HIV older than 50 years who had normal or low BMI.

### Hypertension is associated with the development of Type 2 DM among older people living with HIV

From the literature reviewed, the issue of hypertension was also associated with older people living with HIV who developed Type 2 DM.

In South Africa, Chiwandire et al.^33^ used a sample size of 978 in 2005, 1023 in 2008 and 2483 in 2017 and consistently reported from all three cohorts that age above 45 years and hypertension were predictors of Type 2 DM. The study^33^ used a secondary data analysis method and data from people living with HIV aged more than 25 years from the South African National HIV Prevalence, Incidence, Behaviour and Communication, which is a population-based survey. Duncan, Goff and Peters,^25^ in their 10-year cross-sectional study from 2005 to 2015 in the UK, also found an association between the development of Type 2 DM and comorbidity with hypertension. Duncan, Goff and Peters^25^ defined hypertension with three consecutive readings of more than 140/90 mmHg using an electronic sphygmomanometer or current use of antihypertensives. The authors^25^ from this second older 2015 cohort concluded that the respondents had a higher prevalence of hypertension and Type 2 DM. In Italy, Guaraldi et al.^26^ in their match-control study also discuss that the acquisition of HIV after the age of 50 years is also associated with the development of both Type 2 DM and hypertension. The authors^26^ used guidelines from the European Clinical AIDS Society to define hypertension, which defines hypertension Grade 1 as a reading of 140 mmHg – 159 mmHg/90 mmHg – 99 mmHg.

### Lack of knowledge of modifiable risk factors of Type 2 DM among older people living with HIV is associated with the development of Type 2 DM

The fifth main theme that emerged from the data analysis was that a lack of knowledge on modifiable risk factors for Type 2 DM predisposes older people living with HIV to Type 2 DM. Biraguma et al.,^28^ in a study in Rwanda, concluded that people living with HIV older than 50 years had poor knowledge of the modifiable risks of noncommunicable diseases such as Type 2 DM. The authors^28^ used a sample with a mean age of 38 years and an age range of 18–70 years stratified into four groups: 18–30 years, 31–40 years, 40–50 years and more than 50 years. This sample was drawn from Southern and Northern provinces, which are rural areas, and Kigali City, an urban area. In assessing knowledge levels, Biraguma et al.^28^ used a data collection tool that enquired if respondents had heard of diabetes and knew of risk factors such as smoking, physical inactivity, alcohol use, stress and an unbalanced diet. In a similar study in Tanzania, Kagaruki et al.^27^ found similar results to those found by Biraguma et al.^28^ in Rwanda. The authors^27^ used a sample of 745 people living with HIV from rural Mbeya and urban Dar-es-Salam and found that 42.4% of people living with HIV older than 40 years had low knowledge of modifiable risk factors for developing Type 2 DM. Notably, the study in^27^ Tanzania stratified the sample into two categories: those below 40 years and those above 40 years. In collecting data on knowledge of risks of Type 2 DM, the authors,^27^ like Biraguma et al.,^28^ also assessed knowledge on healthy diet, alcohol use, family history of diabetes and physical exercise.

## Discussion

The scoping review found that the risks associated with the development of Type 2 DM among older people living with HIV include long duration of HIV infection, use of older-generation ART, adverse effects of ART in older people living with HIV, comorbidity with hypertension and a lack of knowledge on modifiable risk factors for Type 2 DM among older people living with HIV. A total of 15 articles were reviewed, which is indicative of the under-representation of research on older people living with HIV at risk of Type 2 DM.

The long duration of HIV infection as a risk factor for the development of Type 2 DM among older people living with HIV was reported throughout all the years the studies were drawn, except in the 2016 study.^35^ Galli et al.^34^ note this long duration of HIV infection as a risk of Type 2 DM in older people living with HIV as early as 2012, through to Høgh et al.^36^ in 2022. Wu et al.^1^ note that HIV was first described in the early eighties, implying that this conclusion of a long duration of HIV infection as a predictor of Type 2 DM in older people living with HIV described by Galli et al.,^34^ shows an ageing population of people living with HIV as early as 2012, who are developing chronic age-related noncommunicable diseases such as Type 2 DM. This also implies that this risk of long duration of HIV as a risk for Type 2 DM is likely to increase, as the ANAC^4^ predicts that 70% of people living with HIV are expected to be more than 50 years by 2030. The theme of the long duration of HIV infection as a risk factor for Type 2 DM among older people was also consistently reported among the different study designs. Five cross-sectional studies,^23,25,26,32,34^ as well as a secondary data analysis study,^24^ a match-control study^29^ and a nonintervention cohort study,^36^ noted the issue of a long duration of HIV infection as a risk of Type 2 DM among older people living with HIV. With regards to the geographical location of the studies, the long duration of HIV infection as a predictor of Type 2 DM among older people living with HIV was also reported in Italy, Zimbabwe, the US, Denmark, the UK and Brazil. The inclusion of the US, where HIV was first described,^1^ also shows an ageing population of people living with HIV. Notably, countries in sub-Saharan Africa (Zimbabwe) where HIV is most prevalent, also reported a long duration of HIV as a risk for Type 2 DM, which could imply an increased burden of HIV and Type 2 DM comorbidity. This implication is reiterated by the WHO,^5^ which reports an increasing number of Type DM in developing countries such as those in sub-Saharan Africa.

Despite nine studies describing the issue of long duration of HIV infection as a risk of Type 2 DM among older people living with HIV, in their descriptions, the definition of long duration of HIV infection that predisposes to Type 2 DM varied. The study in Brazil^30^ noted that the mean duration of the development of Type 2 DM was 10.8 years. An almost similar result was found in Canada,^24^ where it was found that the mean duration to development of Type 2 DM among older people living with HIV was 9 years. The longest duration to the development of Type 2 DM among older people living with HIV was described by Guaraldi et al.,^26^ who described a long duration of HIV infection as longer than 20 years. On the opposite side of the continuum, the study in Zimbabwe^35^ described this long duration of HIV infection to the development of Type 2 DM as more than 7 years.

The effectiveness of ART in the management of HIV has been attributed to the longevity of people living with HIV.^1^ Despite this effectiveness of ART, its adverse effects have also been associated with the development of Type 2 DM.^13^ These adverse effects were described in the second theme to emerge from the study, which was exposure to the older generation of ART, predisposing older people living with HIV to Type 2 DM. Studies^24,26,36^ that described the theme of older-generation ART as a risk of Type 2 DM among older people living with HIV were also consistently drawn from different times: 2016, 2018 and 2022, respectively. This consistency is also maintained through the different study designs applied in the studies, which used secondary data analysis,^24^ a match-control study design^26^ and a nonintervention cohort design.^36^ Notably, the issue of older-generation ART as a predictor of Type 2 DM among older people living with HIV was noted only from studies from Europe and North America. Two of these studies^24,36^ noted the use of stavudine, in particular, in older ART as a predictor of Type 2 DM. Older-generation ART was explicitly explained by Samad et al.,^24^ who outlined that ART initiation between 1997 and 2004 was associated with the development of Type 2 DM. Some scholars such as Høgh et al.^36^ also noted the same; however, they did not explain the exact years that older-generation ART could have been used to predispose older people living with HIV to the development of Type 2 DM.

The occurrence of a high BMI in older people living with HIV was also discussed in four articles.^23,25,26,36^ Studies describing a high BMI as a predictor of Type 2 DM were from different years: two were from 2018,^25,26^ one from 2017^23^ and one from 2022.^36^ Furthermore, the studies were carried out in Europe^25,26^ and Africa.^23^ In addition, different study designs also found that a high BMI among older people living with HIV was associated with Type 2 DM. Two studies^23,25^ used a cross-sectional design, while one study^26^ used a match-control design and the fourth study^36^ used a nonintervention cohort design. These outcomes of high BMI being a predictor of Type 2 DM are consistent with findings by Masenga et al.,^13^ who note that a high BMI predicts Type 2 DM in the general population. In people living with HIV, Farahat et al.^9^ further add that the use of ART results in the redistribution of body fat and weight gain resulting in Type 2 DM. Compounding this, in older people, a decline in body lean mass and an increase in fat mass have also been associated with the development of Type 2 DM.^9^ Despite several scholars noting that a high BMI in older people living with HIV predicts the development of Type 2 DM in older people living with HIV, Samad et al.^24^ found that Type 2 DM does occur in older people living with HIV who have a normal BMI.

The development of hypertension in older people living with HIV also predicted the development of Type 2 DM among older people living with HIV. Three studies^25,26,33^ from the UK, Italy and South Africa noted that the presence of hypertension in older people living with HIV was a predictor of Type 2 DM. Different study designs were used to conclude the findings. The South African Study^33^ used secondary data analysis design, the UK study^25^ used a cross-sectional study design and the Italian study^26^ used a match control study design. The studies were conducted in 2021 (South Africa) and 2018 in the UK and the Italian studies. Notably, the association of developing Type 2 DM and hypertension from the South African study^26^ affirms hypertension’s high prevalence in developing countries, as noted by the WHO.^6^ This further implies the increased burden of hypertension and Type 2 DM among older people living with HIV.

Another risk associated with the development of Type 2 DM among older people living with HIV was a lack of knowledge of modifiable risk factors for the development of Type 2 DM among older people living with HIV. Two studies from Africa^27,28^ noted the issue of the lack of knowledge of modifiable risk factors for the development of Type 2 DM among older people living with HIV. These studies used cross-sectional study designs and were conducted in 2018 and 2019. The finding on the lack of knowledge of modifiable risk factors for the development of Type 2 DM was not saturated because of a lack of representation of the population from other continents. Despite this lack of representation from other continents, the Canadian study^24^ recommended health education on modifiable risk factors as a measure to reduce the incidence of Type 2 DM among older people living with HIV. This recommendation was made despite knowledge of the risks of Type 2 DM not being a variable in the study. This recommendation affirms the findings of these studies.^27,28^

The studies reviewed noted several factors concurrently culminating in the development of Type 2 DM; for instance, Samad et al.^24^ reveal that a long duration of ART is associated with Type 2 DM, which also implies a long duration of HIV infection. Similarly, Høgh et al.^36^ discuss the adverse effects of older-generation thymidine analogues simultaneously with the long duration of HIV infection as a predisposing factor to the development of Type 2 DM among older people living with HIV. Such co-occurrence of several risk factors to the development of Type 2 DM is not unique to older people living with HIV and is also described in studies^10,13^ highlighting the risk factors associated with developing Type 2 DM in the general population. This implies the need for clinicians to holistically assess such risks to identify older people living with HIV at risk of Type 2 DM.

## Conclusion

The study’s objective was to describe risks associated with the development of Type 2 DM among older people living with HIV. From the scoping review of literature, it was concluded that older people living with HIV face excess risks of developing Type 2 DM because of a long duration of HIV infection, having used older-generation ART (including adverse effects of some ART), a high BMI in older people living with HIV the lack of knowledge on modifiable risk factors and the presence of hypertension in older people living with HIV. It is recommended that focused health education should be provided as a means of reducing the incidence of Type 2 DM.

### Limitations

While every effort was made to ensure the accuracy of results reported in this study, the study was limited by the lack of consistency in the definition of Type 2 DM from the studies reviewed. Da Luca et al.^22^ defined Type 2 DM as fasting blood sugar levels above 125 mg/dL or a treating clinician’s diagnosis and use of antidiabetic agents. Han et al.^30^ defined Type 2 DM using any of four criteria: fasting blood sugar of more than or equal to 126 mg/dL or glycated haemoglobin of more than 6.5% or a 2-h fasting plasma oral glucose tolerance test of more than 200 mg/dL or a random plasma glucose more than 200 mg/dL. In two other reviewed studies, Chimbetete et al.^23^ and Samad et al.^24^ defined Type 2 DM as random blood sugar of more than 11.1 mmol/L, confirmed by a fasting blood sugar above 7 mmol/L and symptomatic people living with HIV who had random blood sugar more than 11 mmol/L. Duncan, Goff and Peters^25^ defined Type 2 DM using one criterion of a fasting blood sugar of more than 7 mmol/L. Høgh et al.^36^ defined insulin resistance using homeostasis model assessment – insulin resistance (HOMA-IR).

### Implications

Based on the study findings, clinicians working with older people living with HIV should screen older people living with HIV for these excess risks for developing Type 2 DM and provide health education to reduce the likelihood of developing Type 2 DM. These findings on risks of Type 2 DM among older people living with HIV should also be used by educators teaching Type 2 DM among different population groups. Furthermore, additional research is recommended on the risks of developing Type 2 DM that older people living with HIV face in specific countries to reduce the incidence of Type 2 DM. The risks of developing Type 2 DM among older people living with HIV should also be used by policymakers in the development of guidelines aimed at reducing Type 2 DM.
